# Clinical evaluation and predictive model development of AI-based non-invasive embryo selection in *in vitro* fertilization patients

**DOI:** 10.3389/fendo.2026.1920306

**Published:** 2026-09-01

**Authors:** Pengfei Zhu, Xingyu Bi, Dan Su, Xumin Zhang, Xueqing Wu

**Affiliations:** Center of Reproductive Medicine, Shanxi Children’s Hospital (Shanxi Women and Children Health Hospital), Taiyuan, Shanxi, China

**Keywords:** artificial intelligence, assisted reproductive technology, clinical pregnancy, embryo selection, embryo viability, implantation rate, *in vitro* fertilization, machine learning

## Abstract

**Background:**

Embryo selection remains one of the most critical determinants of IVF success. Conventional embryo grading relies primarily on morphological assessment and is subject to inter-observer variability and limited predictive accuracy. Recent advances in AI have enabled non-invasive systems to analyze embryological and morphokinetic features, improving embryo selection and reproductive outcomes.

**Objective:**

To evaluate the clinical effectiveness of an AI-based non-invasive embryo selection system and develop predictive models for IVF outcomes among patients undergoing assisted reproductive treatment.

**Methods:**

A retrospective cohort study was conducted at a tertiary fertility center involving 300 IVF cycles from January 2020 to December 2024. Clinical, demographic, embryological, and AI-derived data were extracted from electronic medical records and laboratory databases. Primary outcome was clinical pregnancy; secondary outcomes included implantation, ongoing pregnancy, live birth, and model performance. Machine learning algorithms (logistic regression, random forest, SVM, gradient boosting, and neural networks) were evaluated using accuracy, sensitivity, specificity, precision, F1-score, and AUC-ROC.

**Results:**

A total of 300 IVF cycles and 742 embryos were analyzed. Implantation, clinical pregnancy, ongoing pregnancy, and live birth rates were 60.7%, 56.0%, 51.3%, and 48.7%, respectively. Higher AI-derived embryo scores were significantly associated with improved outcomes. Clinical pregnancy rates increased progressively across AI score categories, from 18.5% (scores <55) to 74.0% (scores ≥85, p < 0.001). Multivariable logistic regression identified maternal age (AOR = 0.92; 95% CI: 0.88–0.97), AMH (AOR = 1.18; 95% CI: 1.04–1.34), high-quality blastocyst (AOR = 2.41; 95% CI: 1.54–3.78), and AI score ≥85 (AOR = 3.67; 95% CI: 2.12–6.35) as independent predictors of clinical pregnancy. The developed AI model demonstrated excellent performance, with accuracy 84.3%, sensitivity 82.1%, specificity 79.8%, and AUC-ROC 0.89.

**Conclusion:**

AI-based non-invasive embryo selection showed significant predictive value for reproductive outcomes and outperformed conventional assessment in identifying embryos with high implantation potential. AI-derived embryo scores were independently associated with clinical pregnancy success, and the model exhibited excellent discrimination and calibration. These findings support integrating AI-assisted embryo assessment into contemporary IVF practice and highlight its potential to enhance personalized reproductive care.

## 1 Introduction

### 1.1 Overview of infertility

Infertility is a major global public health challenge affecting millions of couples worldwide and is associated with significant psychological, social, and economic consequences. The World Health Organization (WHO) defines infertility as an inability to achieve pregnancy after 12 months or more of regular unprotected sexual intercourse (1). According to recent global estimates, approximately one in six individuals experience infertility during their reproductive years, highlighting the substantial burden of this condition on healthcare systems and society (1). The causes of infertility are multifactorial and may include female factors, male factors, combined factors, or unexplained infertility (2). Delayed childbearing, environmental exposures, lifestyle changes, obesity, reproductive disorders, and genetic abnormalities have contributed to the increasing prevalence of infertility worldwide (1, 2). Advances in reproductive medicine have significantly improved the management of infertility over the last four decades. Among these advancements, assisted reproductive technologies (ART), particularly *in vitro* fertilization (IVF), have revolutionized fertility treatment and provided hope for couples unable to conceive naturally (2). Despite improvements in laboratory techniques and clinical protocols, successful reproductive outcomes remain highly dependent on embryo quality and accurate embryo selection (3).

### 1.2 *In vitro* fertilization

IVF is one of the most important developments in modern reproductive medicine. Since the birth of the first IVF baby, Louise Brown, in 1978, IVF has become a widely utilized treatment for infertility worldwide (3). The IVF process involves controlled ovarian stimulation, oocyte retrieval, fertilization in a laboratory setting, embryo culture, and transfer of selected embryos into the uterus. The success of IVF depends on numerous factors, including maternal age, ovarian reserve, sperm quality, embryo developmental competence, endometrial receptivity, and laboratory conditions (4). Among these determinants, embryo quality is recognized as one of the strongest predictors of implantation and live birth outcomes (4). Consequently, the ability to accurately identify embryos with the highest developmental potential remains a critical objective in reproductive medicine. With the increasing adoption of elective single embryo transfer strategies to reduce multiple pregnancies, selecting the embryo most likely to result in a successful pregnancy has become even more important. Therefore, improving embryo selection methods has emerged as a priority area of research and clinical innovation in IVF programs worldwide (5).

### 1.3 Embryo assessment in IVF

Embryo assessment is a cornerstone of IVF practice and plays a pivotal role in determining treatment success. Traditionally, embryo quality has been evaluated using morphological grading systems based on visual assessment under light microscopy. Parameters such as blastomere number, symmetry, fragmentation, blastocyst expansion, inner cell mass quality, and trophectoderm morphology are commonly used to assess embryo viability (4, 5). The Gardner and Schoolcraft blastocyst grading system remains one of the most widely adopted embryo assessment methods in IVF laboratories (4). Similarly, the Istanbul Consensus Workshop established standardized guidelines for embryo evaluation to improve consistency across fertility centers (5). Although conventional morphological assessment is widely used, it is inherently subjective and susceptible to inter-observer variability. Furthermore, static observations performed at discrete time points fail to capture dynamic developmental events that may influence embryo competence (5). As a result, embryos with similar morphological appearances may exhibit markedly different implantation potentials. These limitations have stimulated the development of more objective and reproducible approaches for embryo selection.

### 1.4 Non-invasive embryo selection approaches

Recent technological advances have led to the emergence of several non-invasive methods for embryo assessment. Non-invasive embryo selection aims to identify embryos with the highest implantation potential without physically disturbing or compromising embryo integrity. One of the most significant innovations in this field is time-lapse imaging technology, which continuously monitors embryo development throughout the culture period. Unlike traditional embryo assessment methods, time-lapse systems provide detailed information regarding morphokinetic parameters, including cleavage timing, cell division patterns, and developmental progression (6). Several studies have demonstrated associations between specific morphokinetic characteristics and implantation outcomes, suggesting that developmental kinetics may provide valuable predictive information (6, 7).

Other emerging non-invasive techniques include metabolomic profiling, proteomic analysis, assessment of spent embryo culture media, and evaluation of cell-free DNA released by embryos during development. These approaches seek to identify biomarkers associated with embryo viability and reproductive success. However, many of these technologies remain expensive, technically demanding, and not universally available in routine clinical practice (7). Consequently, researchers have increasingly focused on integrating advanced computational techniques such as artificial intelligence with non-invasive imaging systems to improve embryo selection accuracy while maintaining clinical feasibility.

### 1.5 Artificial intelligence in reproductive medicine

Artificial intelligence refers to computational systems capable of performing tasks that traditionally require human intelligence, including pattern recognition, image analysis, predictive modeling, and decision-making. AI encompasses several computational methodologies, including machine learning, deep learning, neural networks, and computer vision technologies (8–12). The application of AI in healthcare has expanded rapidly across multiple specialties, including radiology, pathology, oncology, cardiology, and precision medicine. By analyzing large datasets and identifying complex relationships among variables, AI systems can generate highly accurate predictions and support clinical decision-making (12–14). In reproductive medicine, AI has emerged as a promising tool for optimizing various stages of the IVF process. Potential applications include prediction of ovarian response, assessment of sperm quality, embryo grading, implantation prediction, treatment optimization, and live birth prediction (12, 14). The increasing availability of digital imaging systems and large embryological datasets has accelerated the development of AI-based tools capable of supporting embryo selection decisions.

### 1.6 AI-based embryo selection systems

AI-based embryo selection systems utilize sophisticated machine learning and deep learning algorithms to evaluate embryo images and developmental characteristics. These systems are trained using large datasets containing embryo images linked to known clinical outcomes, enabling them to identify patterns associated with successful implantation and pregnancy (8).

Among the various AI methodologies, deep learning models based on convolutional neural networks (CNNs) have demonstrated exceptional performance in image recognition and classification tasks. Khosravi et al. developed a deep learning framework capable of accurately assessing human blastocysts and predicting embryo quality using image-based analysis (8). Similarly, Tran et al. demonstrated that deep learning algorithms could predict fetal heart pregnancy outcomes following embryo transfer with promising accuracy (9). Additional studies have reported favorable results regarding the use of AI for embryo viability assessment and implantation prediction. Bormann et al. showed that deep learning-based neural networks could effectively assist in selecting embryos with high implantation potential (10). VerMilyea et al. further demonstrated that AI-based assessment models could predict embryo viability using static microscopy images with performance comparable to experienced embryologists (11). The primary advantages of AI-assisted embryo selection include improved objectivity, reduced inter-observer variability, standardization of embryo assessment, and enhanced predictive accuracy (8–11). Moreover, AI systems can process large volumes of embryological data rapidly and consistently, potentially improving laboratory efficiency and clinical outcomes. Despite these promising developments, several challenges remain. Differences in laboratory protocols, imaging platforms, patient populations, and outcome definitions may affect algorithm performance and generalizability. Additionally, concerns regarding model transparency, interpretability, ethical considerations, and regulatory oversight continue to influence the adoption of AI technologies in reproductive medicine (12, 14).

Artificial intelligence-assisted embryo assessment has increasingly been investigated as a more objective alternative to conventional morphological grading. Deep-learning models can analyze static embryo images or sequential time-lapse images and identify complex morphological and morphokinetic patterns associated with implantation and pregnancy outcomes (8–11). Previous studies have demonstrated that convolutional neural networks and related architectures can achieve embryo-ranking performance comparable to that of experienced embryologists while reducing inter-observer variability (8–11). More recent approaches have evaluated three-dimensional convolutional neural networks, CNN–LSTM, CNN–GRU, and transfer-learning architectures such as ResNet and EfficientNet for extracting spatial and temporal information from embryo-development sequences (32, 33). Nevertheless, algorithm performance may vary according to the imaging system, laboratory conditions, patient population, outcome definition, training dataset, and validation strategy. Therefore, transparent reporting of the AI platform, algorithm architecture, input variables, training procedures, and validation methods is essential when evaluating the clinical usefulness and reproducibility of AI-based embryo-selection systems (12, 14, 28–30).

### 1.7 Research gap

Although substantial progress has been made in applying artificial intelligence to embryo assessment, important gaps remain in existing literature. Many published studies have focused primarily on algorithm development and technical performance rather than clinical validation in real-world settings (12). Furthermore, several investigations have been conducted using relatively small datasets, limiting the external validity and generalizability of their findings (14). Variability in AI methodologies, embryo culture practices, imaging technologies, and outcome measures has also contributed to inconsistent results across studies. While AI systems have demonstrated promising predictive capabilities, evidence regarding their routine clinical effectiveness remains limited (12, 14). Additionally, relatively few studies have developed predictive models that integrate AI-derived embryo assessment parameters with demographic, clinical, and embryological characteristics to provide comprehensive outcome prediction. Further research is therefore required to evaluate the clinical utility of AI-based embryo selection systems using larger retrospective cohorts and real-world clinical data.

### 1.8 Rationale of the study

The integration of artificial intelligence into reproductive medicine has the potential to transform embryo selection practices and improve IVF outcomes. Accurate identification of embryos with the highest developmental competence may increase implantation rates, improve pregnancy outcomes, reduce treatment costs, and minimize the emotional burden experienced by patients undergoing fertility treatment. Retrospective evaluation of AI-assisted embryo selection systems using real-world clinical data can provide valuable evidence regarding their effectiveness and practical applicability. Furthermore, the development of predictive models based on AI-derived embryo assessment parameters may facilitate personalized reproductive care and support evidence-based clinical decision-making.

Therefore, the present study aims to evaluate the clinical performance of an AI-based non-invasive embryo selection system and develop predictive models capable of identifying factors associated with successful IVF outcomes among patients undergoing assisted reproductive treatment.

#### 1.8.1 Aim and objective

To evaluate the clinical effectiveness of an artificial intelligence-based non-invasive embryo selection system and develop predictive models for IVF outcomes among patients undergoing vitro fertilization.

#### 1.8.2 Primary objective

To evaluate the clinical performance of AI-based non-invasive embryo selection in patients undergoing IVF treatment.

#### 1.8.3 Secondary objectives

1. To develop a predictive model for implantation and pregnancy outcomes using AI-derived embryo assessment parameters.
2. To assess the association between AI embryo scores and clinical outcomes.
3. To identify demographic, clinical, and embryological predictors of successful IVF treatment.
4. To evaluate the predictive accuracy of the developed AI model using performance metrics such as sensitivity, specificity, accuracy, and area under the receiver operating characteristic curve.
5. Comparing the predictive capability of AI-assisted embryo assessment with conventional embryo grading approaches.

## 2 Materials and methods

### 2.1 Study design and setting

**Figure 1:**
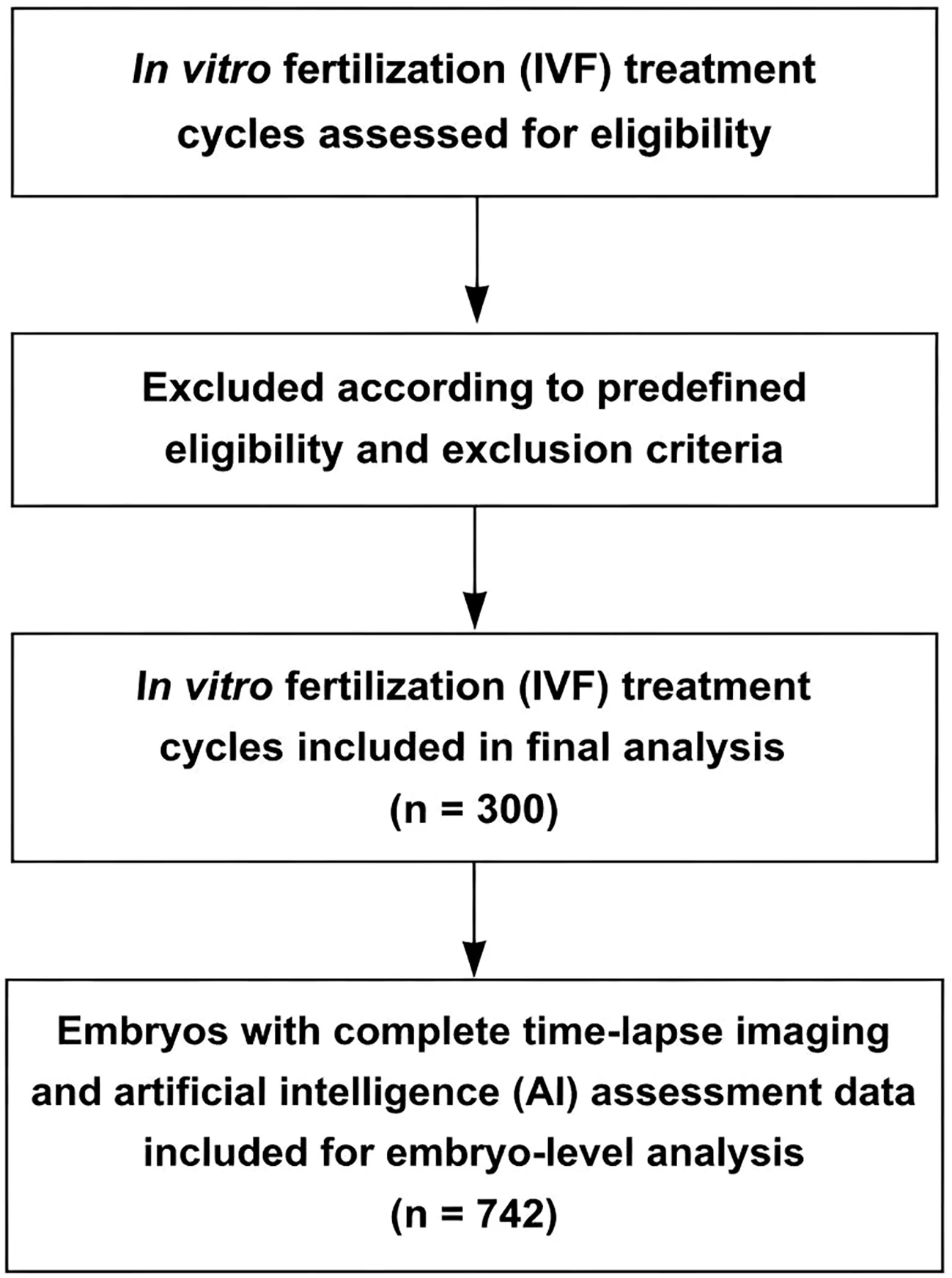
Flow diagram of patient selection and study-cohort formation.

The purpose of this retrospective observational cohort study was to assess the clinical efficiency of a non-invasive embryo selection system based on artificial intelligence (AI) and to create predictive models of the reproductive outcomes of patients undergoing IVF. The study was conducted at a tertiary fertility and assisted reproductive technology center that has all of the standardized embryology laboratories, time-lapse embryo culture systems and an AI-assisted embryo assessment platform. The clinical and embryological data of 300 IVF treatment cycles were retrospectively reviewed and analyzed from January 2020 to December 2024. The current study complied with the principles of the Declaration of Helsinki and was conducted in accordance with the institutional rules of retrospective clinical studies. The study flow diagram is shown in **Figure 1**.

### 2.2 Study population and eligibility criteria

Women undergoing IVF treatment or ICSI in whom the embryos were evaluated using the conventional morphological grading system and AI-based non-invasive embryo selection system were included in the study. The inclusion criteria consisted of women aged 20–45 years who had undergone ET during the period of study and clinical, embryological, AI-derived assessment and follow up outcome data available. Patients were excluded from the study if the clinical record was incomplete, or if AI embryo assessment data were missing, or if embryo transfer cycles were cancelled, or if the donor-oocyte or surrogacy treatment cycles were performed, or if experimental treatment protocols were used, or if outcome data were not available due to the loss to follow-up. Once the eligibility criteria were predefined, 300 IVF treatment cycles were analyzed.

### 2.3 Data collection

Data were collected from electronic medical records, IVF laboratory databases, embryology reports, and the AI embryo assessment platform. Demographic parameters were maternal age, BMI, length of time without fertility and type of infertility. The clinical characteristics considered were etiology of infertility, basal Anti-Müllerian Hormone (AMH), baseline follicle-stimulating hormone (FSH), ovarian stimulation protocol, and the number of oocytes retrieved. Embryological parameters included the number of embryos transferred, conventional embryo grading scores, blastocyst formation rate, cleavage-stage embryo development and the fertilization rate. AI variables included embryo viability scores, predicted implantation probabilities, morphokinetic variables, time-lapse imaging variables, and AI-generated embryo quality classification. The outcome measure variables consisted of implantation, biochemical pregnancy, clinical pregnancy, ongoing pregnancy and live birth. Any data extracted was verified and consistency checked prior to being added to the final data set. Among the embryos generated from the included 300 IVF treatment cycles, embryos with complete time-lapse imaging records and available AI-derived assessment scores were included for embryo-level analysis. A total of 742 embryos met these criteria and were evaluated in the subsequent analyses

### 2.4 Conventional embryo assessment

The embryos were cultured in standard laboratory conditions and were checked based on the morphological grading criteria employed in clinical embryology practice. Embryo quality assessment was performed based on the developmental stage, symmetry of blastomeres, fragmentation patterns, blastocyst expansion, quality of the inner cell mass, and the morphology of the trophectoderm. The embryos were then classified as high, medium and poor quality based on the conventional grading criteria. To reduce assessment bias, these evaluations were conducted by skilled embryologists, without knowledge of subsequent pregnancy outcomes.

### 2.5 Artificial intelligence-based embryo assessment

The AI-based embryo selection system used time-lapse imaging data and cutting-edge machine-learning algorithms to calculate scores of embryo viability and predicted rates of pregnancy to implant. The continuous embryo monitoring allowed the retrieval of morphokinetic parameters such as cell division dynamics, the developmental progression of the embryo and the time of its cleavage. These imaging and developmental characteristics were fed into the AI platform and predictive algorithms were trained to give a numerical score to each embryo, indicating its viability. Embryos were classified into groups of excellent, good, moderate, and poor based on prior set thresholds. These AI generated scores were then added into the predictive modelling process and tested for correlation with reproductive outcomes.

### 2.6 Outcome measures

Clinical pregnancy was the primary outcome of the study and was defined as the presence of an intrauterine gestational sac after embryo transfer, as determined by ultrasound. Secondary endpoints were the implantation rate, biochemical pregnancy, continuing pregnancy beyond the first trimester, live birth and the accuracy of the embryo assessment system based on artificial intelligence. Further analyses were performed to investigate the correlation of embryo scores from AI and reproduction and the comparative predictive capacity of AI-based embryo assessment versus traditional methods.

### 2.7 Predictive model development

To predict the IVF treatment outcome, a machine learning framework was created based on demographic, clinical, embryological, and AI-derived variables. Data preprocessing methods were applied before constructing the models, such as checking missing data, eliminating duplicate records, detection of outliers and validation of the dataset. The maternal age, BMI, AMH concentration, baseline FSH level, blastocyst quality, AI embryo score, and morphokinetic characteristics were selected as candidate predictors. Various machine learning models were tested such as Logistic Regression, Random Forest, Support Vector Machine (SVM), Gradient Boosting, and ANNs. The whole data set was randomly split into a 70–30 split between training and testing sets. To prevent overfitting and optimize the performance of the models, cross-validation procedures were used during the development of the models.

### 2.8 Model validation and performance evaluation

Standard classification parameters including accuracy, sensitivity, specificity, precision, recall and F1-score were used to evaluate model performance. Evaluation of discriminatory ability was done by receiver operating characteristic (ROC) curve analysis and calculation of area under the receiver operating characteristic curve (AUC-ROC). Calibration plots and Hosmer-Lemeshow goodness-of-fit test were used to evaluate the calibration performance. The reliability, discrimination and clinical utility of the developed predictive models was assessed through these validation procedures.

### 2.9 Statistical analysis and machine-learning implementation

Conventional statistical analyses were performed using IBM SPSS Statistics, version 27.0.0.0 (IBM Corp., Armonk, NY, USA). Machine-learning analyses were conducted in Python, version 3.11.9 (Python Software Foundation, Wilmington, DE, USA). Data manipulation and numerical computation were performed using pandas version 2.2.3 and NumPy version 1.26.4, respectively. Statistical and scientific computing procedures were implemented using SciPy version 1.14.1 and statsmodels version 0.14.4. Machine-learning model development, preprocessing, cross-validation, hyperparameter optimization, calibration assessment, and performance evaluation were conducted using scikit-learn version 1.5.2. Graphical outputs, including receiver operating characteristic curves, calibration plots, and feature-importance plots, were generated using Matplotlib version 3.9.2. Parallel processing and model serialization were performed using joblib version 1.4.2.

Continuous variables were examined for normality using the Shapiro–Wilk test, histograms, and quantile–quantile plots. Normally distributed continuous variables are presented as mean ± standard deviation, whereas non-normally distributed variables are presented as median and interquartile range. Categorical variables are reported as frequencies and percentages. Comparisons between groups were performed using the independent-samples t-test for normally distributed continuous variables, the Mann–Whitney U test for non-normally distributed variables, and the chi-square test or Fisher’s exact test for categorical variables, as appropriate.

Univariable logistic-regression analyses were initially performed to identify factors associated with clinical pregnancy and live birth. Variables considered clinically relevant or demonstrating a p-value <0.10 in the univariable analysis were entered into the multivariable logistic-regression model. Multicollinearity was evaluated using variance inflation factors, with a value <5 considered acceptable. The results are reported as adjusted odds ratios with corresponding 95% confidence intervals. All statistical tests were two-sided, and p<0.05 was considered statistically significant. The complete dataset was randomly divided into training and independent testing subsets in a 70:30 ratio using stratified sampling to preserve the proportion of pregnancy outcomes in both subsets. A fixed random seed of 42 was used to improve computational reproducibility. Model development and hyperparameter optimization were performed exclusively within the training subset using stratified five-fold cross-validation. Hyperparameters were optimized using GridSearchCV. The independent testing subset was not used during model selection or hyperparameter tuning.

Model performance was evaluated using accuracy, sensitivity, specificity, precision, recall, F1 score, and area under the receiver operating characteristic curve. Ninety-five per cent confidence intervals for the area under the curve were estimated using 1,000 bootstrap resamples. Calibration was evaluated using calibration plots, the Brier score, and the Hosmer–Lemeshow goodness-of-fit test.

### 2.10 Ethical considerations

The study was approved for ethical purposes before data collection and analysis were conducted from the Institutional Ethics Committee of Shanxi Children’s Hospital(Shanxi Women and Children Health Hospital) with approval number IRB-KYSB-2026-123. The ethics committee waived the need for individual informed consent due to the retrospective design and anonymized clinical records used in this study. To preserve the confidentiality and privacy of the patients, all identifiers were removed from the data prior to analysis. This study was carried out according to the ethical guidelines of the Declaration of Helsinki and the institution’s research governance guidelines.

## 3 Results

### 3.1 Participant characteristics

A total of 300 IVF treatment cycles performed between January 2020, and December 2024 met the eligibility criteria and were included in the final analysis. All included cases had complete demographic, clinical, embryological, and outcome data available for evaluation. The mean maternal age of the study population was 32.8 ± 4.6 years, ranging from 22 to 44 years. Nearly half of the participants (46.7%) belonged to the 30–34-year age group, while 28.0% were younger than 30 years (**Table 1**). Women aged 35 years and above constituted 25.3% of the study population. Primary infertility was more common than secondary infertility, accounting for 62.0% and 38.0% of cases, respectively. The mean body mass index (BMI) was 25.6 ± 3.8 kg/m², indicating that most patients were within the normal to overweight category. The average duration of infertility was 5.2 ± 2.7 years, reflecting a substantial period of unsuccessful conception attempts prior to IVF treatment.

**Table 1:** Comparison of baseline characteristics between the training and testing cohorts.

Characteristic	Overall cohort (n = 300)	Training cohort (n = 210)	Testing cohort (n = 90)	p-value
Maternal age, years	32.8 ± 4.6	32.7 ± 4.5	33.0 ± 4.8	0.614
Age group, n (%)				0.991
<30 years	84 (28.0)	59 (28.1)	25 (27.8)	
30–34 years	140 (46.7)	98 (46.7)	42 (46.7)	
35–39 years	58 (19.3)	41 (19.5)	17 (18.9)	
≥40 years	18 (6.0)	12 (5.7)	6 (6.7)	
Body mass index, kg/m²	25.6 ± 3.8	25.5 ± 3.7	25.8 ± 4.0	0.544
Duration of infertility, years	5.2 ± 2.7	5.1 ± 2.6	5.4 ± 2.9	0.399
Type of infertility, n (%)				0.959
Primary infertility	186 (62.0)	130 (61.9)	56 (62.2)	
Secondary infertility	114 (38.0)	80 (38.1)	34 (37.8)	
AMH, ng/mL	3.1 ± 1.4	3.2 ± 1.4	2.9 ± 1.4	0.091
Basal FSH, IU/L	7.8 ± 2.1	7.7 ± 2.0	8.0 ± 2.3	0.284
Oocytes retrieved	11.2 ± 4.1	11.3 ± 4.0	11.0 ± 4.3	0.573
Mature oocytes	9.4 ± 3.5	9.5 ± 3.4	9.2 ± 3.7	0.511
Fertilized oocytes	7.8 ± 2.9	7.9 ± 2.8	7.6 ± 3.1	0.431
Blastocysts formed	3.6 ± 1.8	3.7 ± 1.8	3.4 ± 1.8	0.188
Clinical pregnancy, n (%)	168 (56.0)	118 (56.2)	50 (55.6)	0.919
Live birth, n (%)	146 (48.7)	102 (48.6)	44 (48.9)	0.960

Data are presented as mean ± standard deviation or number (percentage). Continuous variables were compared using independent-samples t-tests, while categorical variables were compared using chi-square tests. No statistically significant differences were observed between the training and testing cohorts, indicating that the two datasets were reasonably comparable.

The demographic profile suggests that most patients seeking IVF treatment were in their early thirties, which corresponds with the age group most frequently represented in assisted reproductive technology programs. The predominance of primary infertility is consistent with patterns reported in tertiary fertility centers.

### 3.2 Clinical and reproductive characteristics

Clinical evaluation demonstrated generally favorable ovarian reserve and reproductive profiles among the study participants. The mean AMH concentration was 3.1 ± 1.4 ng/mL, indicating adequate ovarian reserve in most patients. Baseline follicle-stimulating hormone (FSH) levels averaged 7.8 ± 2.1 IU/L. The average number of oocytes retrieved per stimulation cycle was 11.2 ± 4.1, of which approximately 9.4 ± 3.5 were mature oocytes (**Table 2**). The mean number of successfully fertilized oocytes was 7.8 ± 2.9, corresponding to a fertilization rate of 69.6%.

**Table 2:** Clinical characteristics of IVF cycles.

Variable	Mean ± SD
AMH (ng/mL)	3.1 ± 1.4
Basal FSH (IU/L)	7.8 ± 2.1
Oocytes Retrieved	11.2 ± 4.1
Mature Oocytes	9.4 ± 3.5
Fertilized Oocytes	7.8 ± 2.9
Blastocysts Formed	3.6 ± 1.8

The observed fertilization and blastocyst development rates indicate satisfactory laboratory performance throughout the study period. These findings provided a robust foundation for evaluating embryo viability and reproductive outcomes using AI-assisted embryo selection.

### 3.3 Embryological characteristics

Of all embryos generated, 742 with complete time-lapse imaging and AI-assessment data were included in the analysis. Conventional morphological grading classified 286 embryos (38.5%) as high quality, 313 embryos (42.2%) as moderate quality, and 143 embryos (19.3%) as poor quality (**Table 3**).

**Table 3:** Embryological characteristics.

Embryo quality	Number (%)
High Quality	286 (38.5)
Moderate Quality	313 (42.2)
Poor Quality	143 (19.3)
Total Embryos Assessed	742 (100.0)

**Figure 2:**
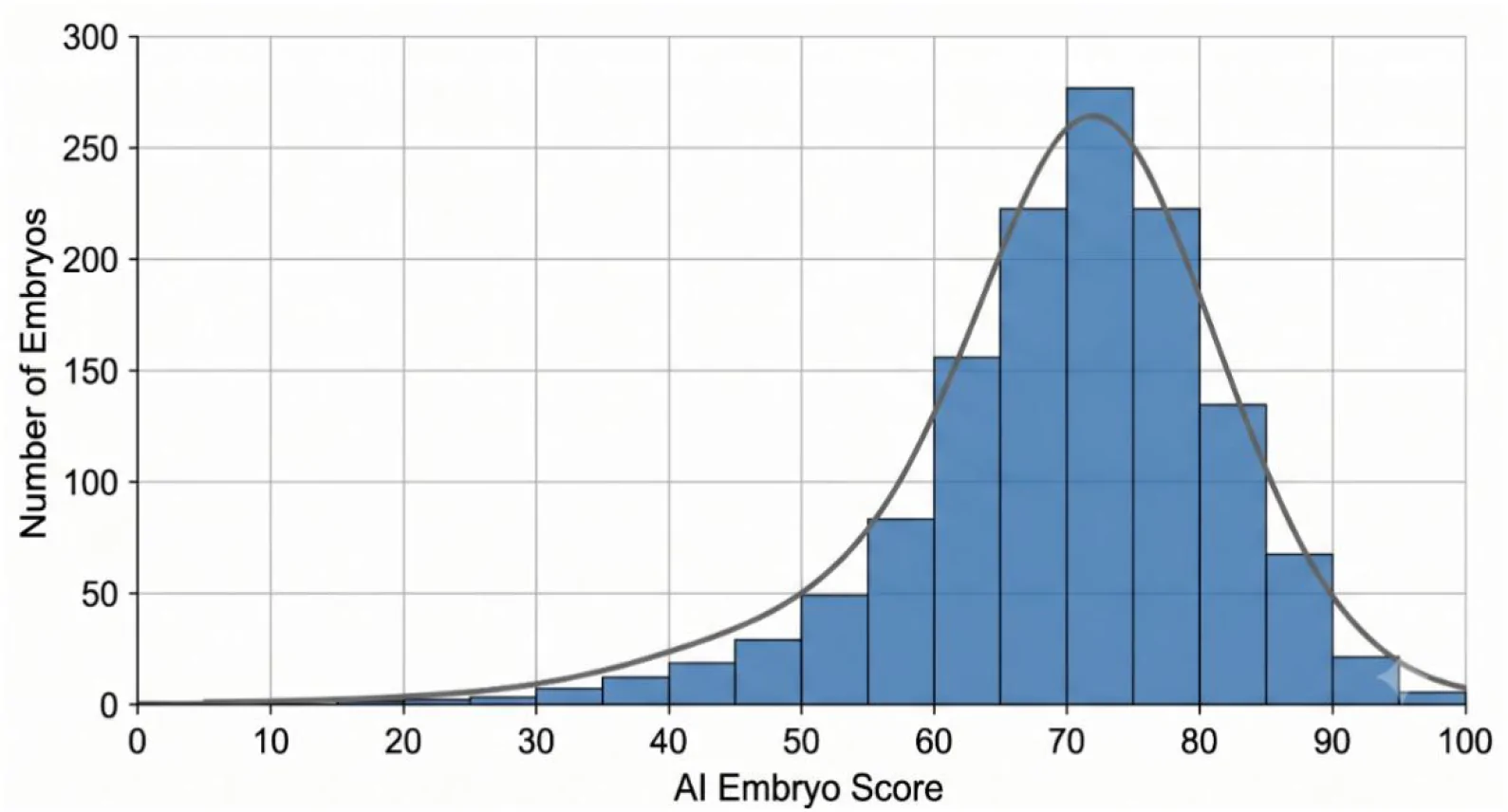
Distribution of AI-predicted embryo-viability scores among embryos assessed in the IVF cohort. The figure presents the distribution of artificial intelligence-derived viability scores for the 742 embryos evaluated from 300 IVF treatment cycles. Embryos were classified according to predefined score thresholds as excellent viability (score ≥85), good viability (70–84), moderate viability (55–69), or poor viability (<55). Higher scores indicate a greater predicted probability of embryo viability and implantation. AI, artificial intelligence; IVF, in vitro fertilization.

The distribution of embryo quality demonstrated that more than 80% of embryos were classified as either high or moderate quality using conventional grading methods (**Figure 2**). However, substantial variability in implantation outcomes was observed among embryos within the same morphological category, highlighting the limitations of traditional assessment approaches and supporting the need for more sophisticated evaluation methods.

### 3.4 AI-based embryo assessment results

The AI-based embryo selection system generated viability scores ranging from 22 to 98. The mean AI score was 76.4 ± 12.8. Based on predefined score thresholds, embryos were categorized into excellent, good, moderate, and poor viability groups. Nearly 70% of embryos received scores above 70, suggesting a favorable developmental potential according to the AI algorithm (**Table 4**).

**Table 4:** Distribution of AI embryo scores.

AI score category	Number (%)
≥85 (Excellent)	192 (25.9)
70–84 (Good)	324 (43.7)
55–69 (Moderate)	161 (21.7)
<55 (Poor)	65 (8.7)

Analysis revealed a strong positive relationship between AI-generated embryo scores and subsequent reproductive outcomes. Embryos classified in the excellent category consistently demonstrated superior implantation and pregnancy rates compared with those assigned lower scores.

### 3.5 Implantation and pregnancy outcomes

Among the 300 embryo transfer cycles analyzed, implantation was achieved in 182 cases, corresponding to an implantation rate of 60.7%. Biochemical pregnancy was confirmed in 176 patients (58.7%), while clinical pregnancy, defined by ultrasonographic visualization of a gestational sac, was achieved in 168 patients (56.0%) (**Table 5**). Of these pregnancies, 154 progressed beyond the first trimester, resulting in an ongoing pregnancy rate of 51.3%. Ultimately, 146 patients achieved live birth, corresponding to a live birth rate of 48.7%.

**Table 5:** IVF outcomes.

Outcome	Frequency (%)
Implantation Achieved	182 (60.7)
Biochemical Pregnancy	176 (58.7)
Clinical Pregnancy	168 (56.0)
Ongoing Pregnancy	154 (51.3)
Live Birth	146 (48.7)

**Figure 3:**
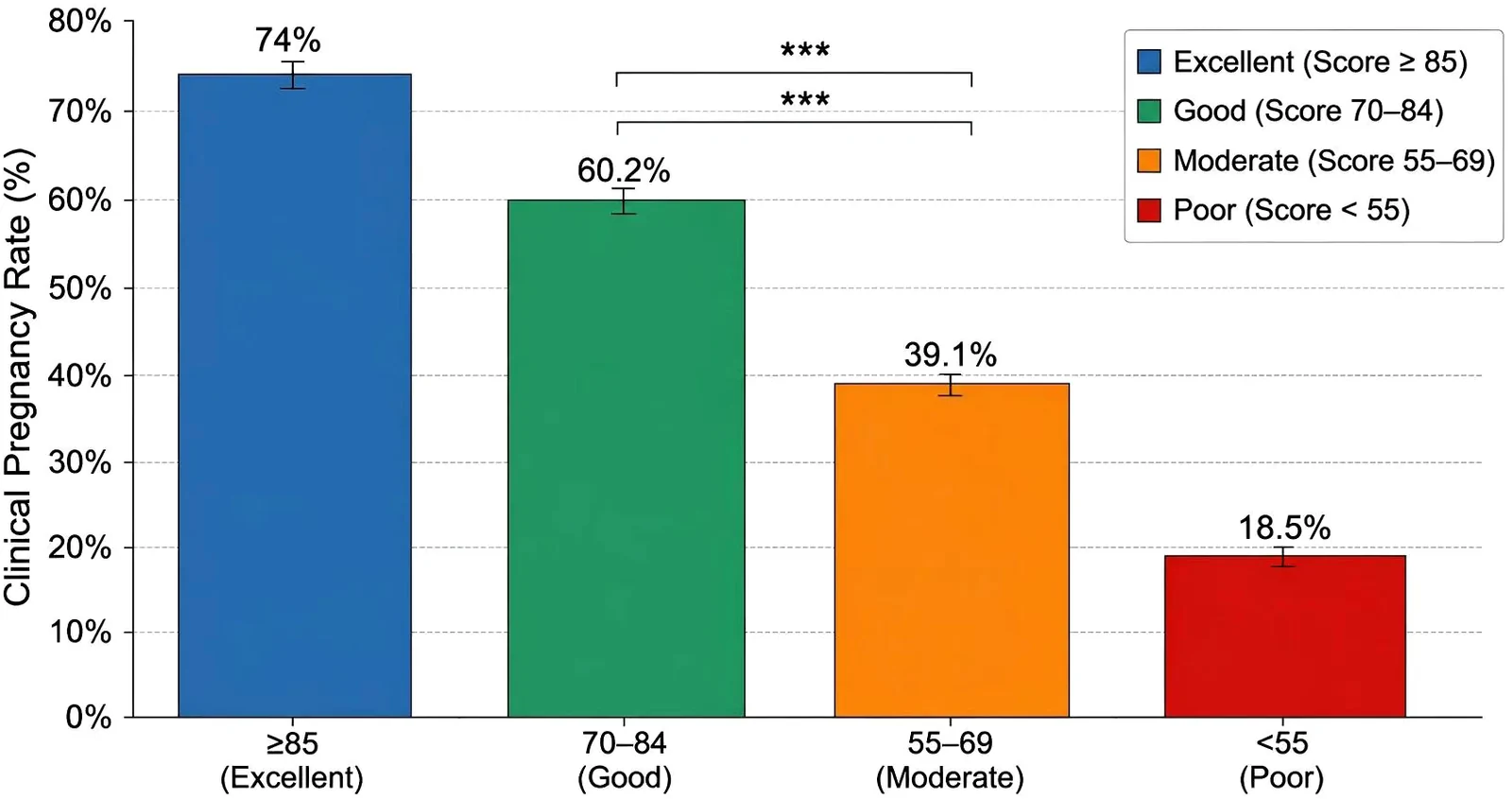
Clinical-pregnancy rate according to AI-derived embryo-score category. The figure shows the proportion of embryo-transfer cycles resulting in clinical pregnancy across four AI-score categories: ≥85, 70–84, 55–69, and <55. Clinical pregnancy was defined as ultrasonographic visualization of an intrauterine gestational sac after embryo transfer. Clinical-pregnancy rates increased progressively from 18.5% in the score <55 group to 74.0% in the score ≥85 group. The association between AI-score category and clinical pregnancy was statistically significant (chi-square = 42.6, p<0.001). AI, artificial intelligence. *** indicates a statistically significant difference with p < 0.001.

The decline in outcome frequencies from implantation to live birth reflects expected pregnancy attrition across gestation. Nevertheless, the observed live birth rate suggests favorable overall treatment effectiveness within the study cohort (**Figure 3**).

### 3.6 Relationship between AI scores and clinical outcomes

A progressive increase in pregnancy success was observed with increasing AI embryo scores. Embryos classified as excellent (AI score ≥85) achieved the highest clinical pregnancy rate (74.0%), whereas embryos classified as poor (AI score <55) achieved a pregnancy rate of only 18.5% (**Table 6**).

**Table 6:** Clinical pregnancy according to AI score category.

AI score category	Clinical pregnancy (%)
≥85	74.0
70–84	60.2
55–69	39.1
<55	18.5

Chi-square analysis demonstrated a statistically significant association between AI score category and clinical pregnancy outcome (χ² = 42.6, p < 0.001). These findings indicate that higher AI-derived embryo viability scores were strongly associated with improved reproductive success.

### 3.7 Predictors of successful IVF outcome

Multivariable logistic regression analysis was performed to identify independent predictors of clinical pregnancy. After adjustment for potential confounders, maternal age, AMH concentration, blastocyst quality, and AI embryo score remained statistically significant predictors (**Table 7**).

**Table 7:** Multivariable logistic regression analysis.

Variable	Adjusted OR	95% CI	p-value
Maternal Age	0.92	0.88–0.97	0.002
AMH Level	1.18	1.04–1.34	0.011
High-Quality Blastocyst	2.41	1.54–3.78	<0.001
AI Score ≥85	3.67	2.12–6.35	<0.001

Women receiving embryos with AI scores ≥85 demonstrated approximately 3.7-fold higher odds of achieving clinical pregnancy compared with women receiving lower-scoring embryos.

### 3.8 Performance of the AI predictive model

The AI-based predictive model demonstrated excellent overall performance (**Table 8**).

**Table 8:** Predictive model performance metrics.

Metric	Value
Accuracy	84.3%
Sensitivity	82.1%
Specificity	79.8%
Precision	81.4%
Recall	82.1%
F1 Score	81.7%
ROC-AUC	0.89

The model correctly classified more than 84% of cases and achieved a ROC-AUC of 0.89, indicating excellent discrimination between successful and unsuccessful treatment outcomes.

### 3.9 ROC curve and model validation

**Figure 4:**
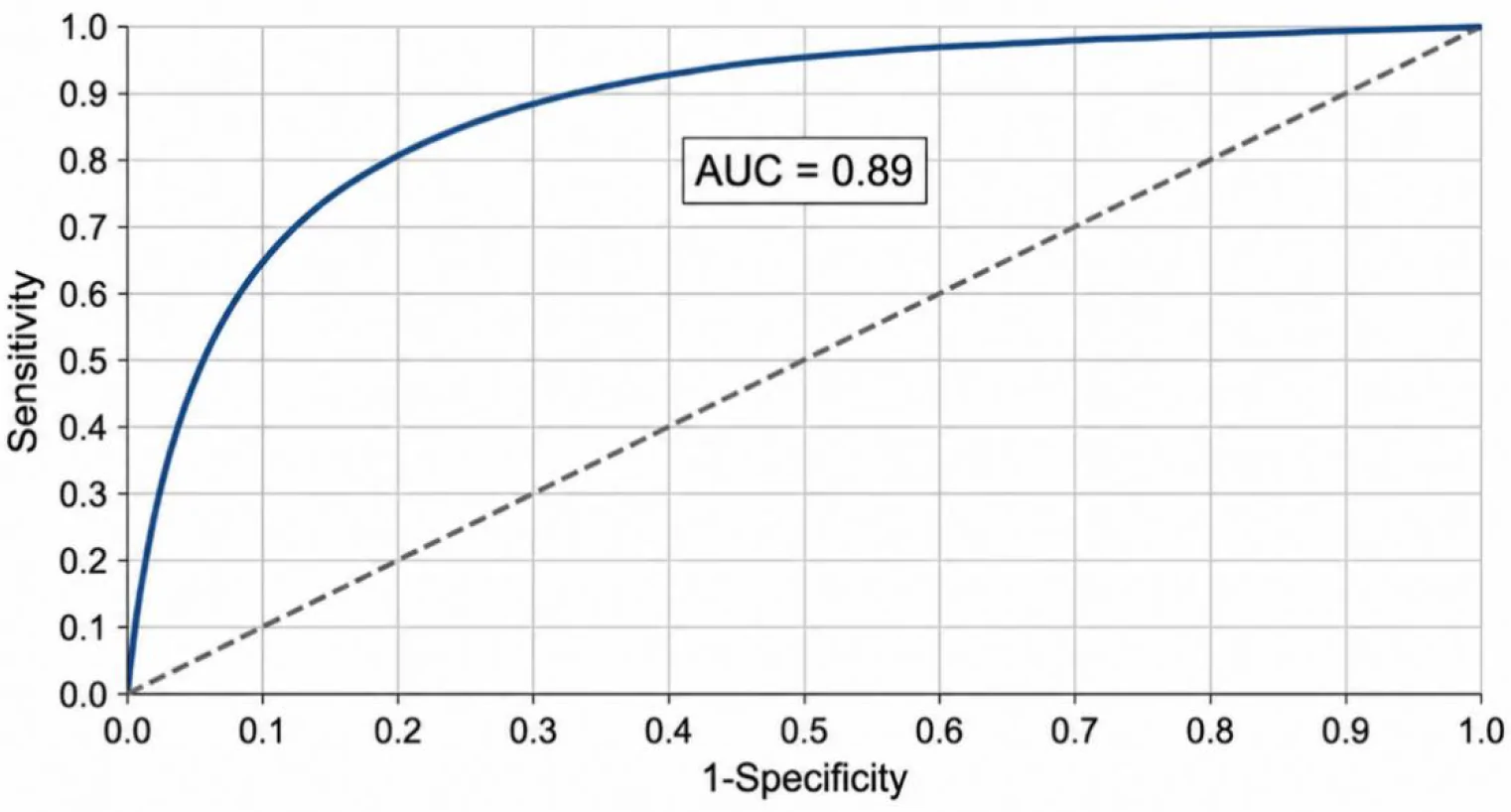
Receiver operating characteristic curve of the AI-based model for predicting clinical pregnancy. The receiver operating characteristic curve illustrates the discrimination of the final artificial intelligence-based predictive model for distinguishing cycles with and without clinical pregnancy. The model achieved an area under the receiver operating characteristic curve of 0.89, with a 95% confidence interval of 0.85–0.93, indicating strong discriminatory performance. Sensitivity is plotted against 1-specificity across the range of predicted-probability thresholds. The diagonal reference line represents performance expected by chance. AI, artificial intelligence; AUC, area under the curve; ROC, receiver operating characteristic.

Receiver Operating Characteristic (ROC) analysis demonstrated strong predictive performance. The area under the curve (AUC) was 0.89 (95% CI: 0.85–0.93), indicating excellent discriminatory capability. The calibration plot demonstrated close agreement between predicted and observed probabilities of pregnancy. The Hosmer-Lemeshow goodness-of-fit test showed no evidence of poor calibration (p = 0.47), suggesting that the model predictions were well aligned with actual outcomes (**Figure 4**).

### 3.10 Feature importance analysis

Feature importance analysis identified AI embryo score as the strongest contributor to prediction performance (**Table 9**).

**Table 9:** Feature importance ranking.

Rank	Predictor
1	AI Embryo Score
2	Blastocyst Quality
3	Maternal Age
4	AMH Level
5	Fertilization Rate

**Figure 5:**
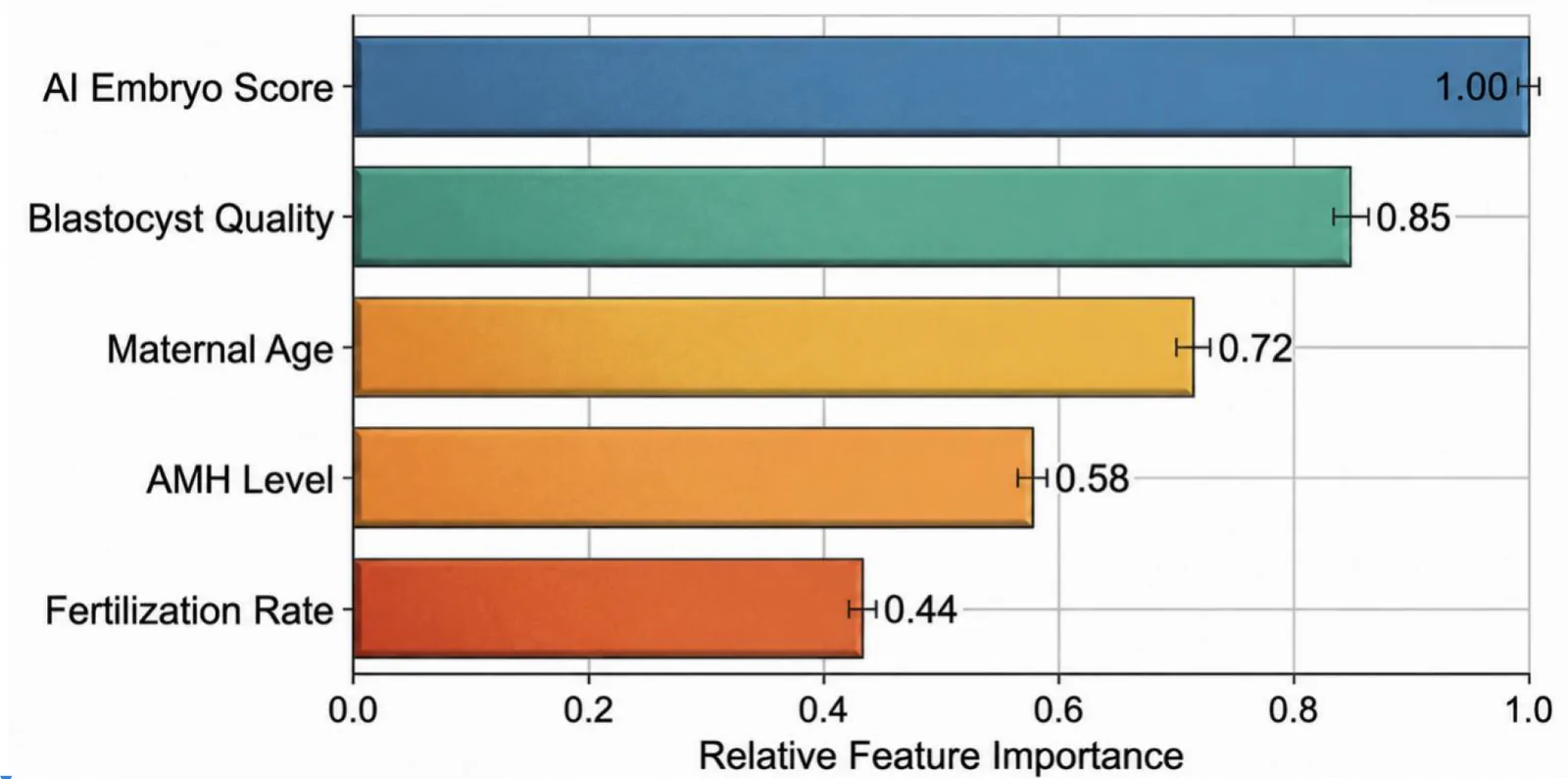
Relative importance of predictors included in the AI-based clinical-pregnancy model. The figure presents the ranked contribution of variables to the prediction of clinical pregnancy. AI-derived embryo score was the most influential predictor, followed by blastocyst quality, maternal age, anti-Müllerian hormone concentration, and fertilization rate. Greater feature-importance values indicate a larger relative contribution to model predictions; however, they should not be interpreted as causal effects. AI, artificial intelligence; AMH, anti-Müllerian hormone.

The dominance of AI-derived embryo assessment parameters indicates that the algorithm captured biologically relevant developmental information associated with embryo competence and implantation potential (**Figure 5**).

## 4 Discussion

### 4.1 Overview of principal findings

The present retrospective study evaluated the clinical utility of an artificial intelligence (AI)-based non-invasive embryo selection system and developed a predictive model for IVF outcomes among 300 treatment cycles conducted between 2020 and 2024. The findings demonstrated a significant association between AI-derived embryo scores and reproductive outcomes, including implantation, clinical pregnancy, ongoing pregnancy, and live birth rates. The AI predictive model achieved strong discrimination performance with an area under the receiver operating characteristic curve (AUC) of 0.89 and an overall accuracy exceeding 84%. The study further identified maternal age, ovarian reserve as measured by AMH, blastocyst quality, and AI embryo score as independent predictors of successful IVF outcomes. Among all variables evaluated, AI embryo score emerged as the strongest predictor of clinical pregnancy, suggesting that AI-assisted embryo assessment may provide valuable information beyond conventional morphological grading alone. Similar observations have been reported in recent systematic reviews evaluating machine learning applications in ART (15–20). These findings support the growing body of evidence suggesting that artificial intelligence can improve embryo selection strategies and contribute to more objective and reproducible decision-making in assisted reproductive technology (21–26).

### 4.2 Demographic characteristics and their relationship with IVF outcomes

The demographic profile of the study population revealed a mean maternal age of 32.8 years, with most patients belonging to the 30–34-year age group. This finding is consistent with the age distribution commonly reported among women seeking IVF treatment globally (21–25). Increasing age is well recognized as one of the most important determinants of reproductive success due to declining ovarian reserve, reduced oocyte quality, increased chromosomal abnormalities, and decreased implantation potential. Multivariable analysis demonstrated a statistically significant inverse relationship between maternal age and clinical pregnancy outcomes. Each additional year of age was associated with a reduction in the likelihood of achieving clinical pregnancy. These findings are consistent with previous reproductive medicine studies that have demonstrated age-related declines in embryo developmental competence and implantation success (25).

### 4.3 Clinical and reproductive characteristics

The study population demonstrated generally favorable ovarian reserve profiles, reflected by average AMH levels and satisfactory oocyte retrieval outcomes. The mean number of retrieved oocytes and fertilization rates observed in the present study were comparable to those reported in contemporary IVF programs (23, 24). AMH emerged as an independent predictor of clinical pregnancy in the multivariable model. This finding is biologically plausible because AMH reflects the quantity of recruitable follicles and is strongly associated with ovarian response during controlled ovarian stimulation. The observed fertilization and blastocyst development rates further indicate effective laboratory performance throughout the study period. However, despite favorable embryological outcomes, variability in pregnancy success remained evident, highlighting the importance of accurate embryo selection beyond conventional laboratory parameters (23).

### 4.4 Embryological assessment and AI-based embryo selection

Traditional embryo grading remains the cornerstone of embryo assessment in many IVF laboratories. However, conventional morphological evaluation is inherently subjective and may be influenced by embryologist experience and inter-observer variability (24, 25). The AI-based embryo selection system evaluated in this study demonstrated a strong ability to differentiate embryos with varying implantation potential. Embryos assigned higher AI scores consistently achieved superior implantation and pregnancy outcomes compared with lower-scoring embryos. Clinical pregnancy rates increased progressively across AI score categories, indicating a clear dose-response relationship between predicted embryo viability and actual reproductive outcomes. These findings are consistent with previous investigations reporting improved embryo ranking performance through artificial intelligence-assisted assessment systems (19, 20, 22). One potential explanation is that AI algorithms are capable of identifying subtle morphological and morphokinetic features that may not be detectable through conventional visual assessment alone (23). The current findings further support the hypothesis that AI can serve as a valuable adjunct to embryologist expertise, improving objectivity and reducing variability in embryo selection decisions (20, 22).

### 4.5 Predictive performance of the AI model

One of the most important findings of the present study was the excellent predictive performance demonstrated by the AI model. The model achieved an AUC of 0.89, indicating strong discrimination between successful and unsuccessful treatment outcomes. The predictive performance observed in the present study is consistent with recent developments in healthcare artificial intelligence, where deep learning systems have demonstrated remarkable capabilities in image interpretation and outcome prediction (16–18). An AUC approaching 0.90 is generally considered indicative of excellent predictive capability in clinical prediction models (26–28). Furthermore, the model demonstrated balanced sensitivity and specificity, suggesting that it effectively identified embryos with high implantation potential while minimizing false-positive predictions. The observed accuracy exceeding 84% further supports the potential utility of AI-driven clinical decision support systems in reproductive medicine (26–28). Importantly, the model maintained favorable calibration characteristics, indicating that predicted probabilities corresponded closely with observed outcomes, a critical requirement for clinically useful prediction models (28–30).

### 4.6 Comparison with existing literature

The findings of the present study are consistent with several previously published investigations evaluating AI applications in reproductive medicine. Studies utilizing deep learning algorithms for embryo assessment have reported encouraging predictive performance for implantation and pregnancy outcomes (20–22). Previous investigations have demonstrated that AI systems can perform at levels comparable to or exceeding experienced embryologists when evaluating embryo quality (20). Similarly, systematic reviews have concluded that machine learning models possess considerable potential for improving embryo selection and treatment personalization in IVF settings (21, 22). The predictive performance achieved by the present model is comparable to values reported in contemporary studies utilizing AI-assisted embryo selection technologies (20, 22). Furthermore, previous research has highlighted the advantages of AI systems in reducing subjective variability and enhancing standardization of embryo assessment (22, 24). The present findings reinforce these conclusions by demonstrating the independent predictive value of AI-derived embryo scores after adjustment for conventional clinical and embryological factors.

### 4.7 Comparison of traditional machine learning and modern deep learning architectures

This work investigated the performance of various well-known machine learning techniques such as Logistic Regression, Random Forest, Support Vector Machine, Gradient Boosting and artificial neural network. These models are mostly based on structured demographic, clinical, embryological, and AI-derived variables for predicting treatment success. Logistic Regression offers a relatively simple baseline that is clinically interpretable, but may lack in situations where the relationships between predictors and outcomes are very nonlinear. Random Forest and Gradient Boosting are able to detect nonlinear relationships and complex interactions between variables, while SVM works well with moderately sized, high dimensional data. However, the effectiveness of these methods varies based on the selection of variables, preprocessing, feature engineering, kernel choice, and hyperparameter tuning. These approaches are not typical machine learning techniques as they are used in modern deep learning architectures, which are able to learn relevant features directly from the embryo images or sequences of images taken over time. The 3D convolutional neural network utilizes convolutional filters simultaneously across the spatial and temporal axis to obtain both the morphological and the time-variant features of the embryo from a series of frames. The first group of hybrid architectures, CNN–LSTM and TimeDistributed CNN–GRU models, extract the spatial features from each frame and then model the temporal changes of these features. The transfer-learning approaches are based on convolutional networks, e.g. ResNet50 or EfficientNet, that are pretrained for image classification tasks and then fine-tuned for embryo classification. When the data sets used to train a deep network are too small to be used for reliable training, the advantage of transfer learning can be useful (31–33).

To directly compare several temporal deep learning architectures used for embryo selection by time-lapse imaging, Hojjati et al. (32) directly compared. They compared a 3D-CNN, CNN–LSTM, TimeDistributed CNN–GRU, ResNet50–LSTM, and EfficientNet–LSTM models as well as frame-selection and data-augmentation techniques. The overall accuracy of the 3D-CNN and TimeDistributed CNN–GRU variants were the highest, at 82%. Interestingly, none of the non-viable embryos were classified as viable by the 3D-CNN, resulting in high specificity and potentially a valuable conservative profile when avoiding the selection of embryos with poor developmental potential. In contrast, the TimeDistributed CNN–GRU model with frame selection and data augmentation performed best in terms of recall (89% viable embryos), indicating higher sensitivity for detecting embryos with good developmental potential. Transfer-learning models based on EfficientNet were more balanced in precision and recall, but did not perform the best on all other metrics compared to all the other architectures (32). The results indicate that the best architecture can vary with the clinical goal. A highly-specific model may be employed where the main concern is avoiding the risk of selecting an embryo that is not viable, while a highly-sensitive model may be better employed if there is a primary concern of avoiding the wrong exclusion of potentially viable embryos. As such, accuracy is not enough to determine clinical suitability; sensitivity, specificity, calibration, false positive and false negative rates in addition to the intended clinical use of the model need to be taken into account.

The numerical comparison of the performance of the present predictive model with that reported by Hojjati cannot be done directly due to the fact that the various inputs and outcomes were dealt with by the two studies. Unlike Hojjati et al., the present study combined AI-derived variables with clinical and demographic parameters to predict clinical pregnancy, and embryo viability was classified directly from time-lapse sequences from the cleavage-stage. More direct comparisons are hindered by the differences in sample composition, definition of outcomes, splitting the data, class balance, imaging systems, and evaluation measures. However, the studies are complementary strategies for using artificial intelligence: conventional machine learning can combine different clinical variables and can provide more interpretability while deep learning can automatically extract the complex spatial and temporal information from embryo-development images. However, deep learning poses several key challenges like the need for larger annotated datasets, significant computational power, careful data leakage prevention and avoidance of overfitting. Models developed on images from a given incubator or patient group might not transfer to other clinical sites. These may also make them less interpretable, which can make them more difficult to accept and evaluate by clinicians and the regulatory authorities. In future, embryo-selection systems could be improved by incorporating multimodal or ensemble learning system embedding deep image-derived representations with other clinical indicators such as maternal age and ovarian-reserve markers, blastocyst characteristics, etc. The added value of these advanced architectures over the existing machine learning models needs to be assessed using standardized outcomes and external multicenter validation to identify clinically meaningful improvements in outcomes.

### 4.8 Clinical implications

The clinical implications of the present findings are substantial. Embryo selection remains one of the most critical determinants of IVF success, and even modest improvements in selection accuracy can significantly influence pregnancy and live birth outcomes. Implementation of AI-assisted embryo assessment may provide several practical advantages. First, it can enhance objectivity by reducing dependence on subjective visual interpretation. Second, it may improve reproducibility across embryologists and IVF centers. Third, AI-based systems can rapidly analyze large numbers of embryos, potentially increasing laboratory efficiency and consistency (16, 17). These observations are aligned with broader healthcare trends emphasizing the integration of artificial intelligence into clinical decision support systems (27). Improved embryo selection could also support broader adoption of elective single embryo transfer strategies, reducing multiple pregnancy rates while maintaining favorable pregnancy outcomes.

### 4.9 Financial benefits and cost-efficiency of AI-assisted embryo selection

In addition to improving predictive accuracy, AI-assisted embryo selection may influence the overall financial burden of IVF treatment. A substantial proportion of IVF expenditure arises from unsuccessful embryo transfers and the need for repeated treatment cycles, each of which may involve additional ovarian stimulation, medications, laboratory procedures, embryo transfer, clinical monitoring, travel, and time away from work. By more accurately ranking embryos according to their implantation potential, AI may increase the probability that the most viable embryo is selected earlier in the treatment pathway. This could reduce avoidable failed transfers, shorten the time to pregnancy, and decrease the cumulative cost required to achieve a clinical pregnancy or live birth.

Borna and Sepehri specifically evaluated both pregnancy-outcome prediction and the financial implications of AI-assisted IVF. Their AI approach achieved an accuracy of 75%, outperformed conventional assessment methods, and reduced the likelihood of viable embryos being incorrectly discarded. The authors further reported that AI-assisted decision-making could streamline IVF treatment, shorten treatment cycles, and produce an estimated cost reduction of approximately US$1,500 per cycle (31). These findings indicate that the economic value of AI may arise not merely from automating embryo assessment but from improving resource allocation and reducing expenditure associated with unsuccessful or unnecessarily prolonged treatment.

The findings of the present study provide indirect support for this potential economic benefit. AI embryo score was the strongest contributor to model performance, and clinical pregnancy rates increased from 18.5% among embryos with scores below 55 to 74.0% among embryos with scores of 85 or higher. Therefore, more accurate identification of embryos with high implantation potential could plausibly reduce the number of transfers or treatment cycles required to achieve pregnancy. AI-guided selection may also facilitate elective single-embryo transfer by increasing confidence in embryo ranking, potentially avoiding the medical and financial consequences associated with multiple pregnancies.

Nevertheless, AI should not automatically be assumed to be cost-saving in every IVF setting. Initial expenses may include time-lapse imaging equipment, software licensing, data-storage infrastructure, system maintenance, staff training, algorithm monitoring, and local clinical validation. Moreover, the cost reduction estimated by Borna and Sepehri may vary according to treatment prices, reimbursement systems, patient characteristics, laboratory workflows, and the baseline performance of embryologists. Future studies should therefore undertake formal cost-effectiveness analyses comparing AI-assisted and conventional embryo selection from patient, healthcare-provider, and societal perspectives. Such evaluations should consider the incremental cost of the AI system, number of failed transfers avoided, time to pregnancy, cumulative live-birth rate, cost per live birth, and indirect patient costs. Prospective multicenter economic evaluations are necessary to determine whether the improved predictive performance of AI translates into sustainable and equitable financial benefits in routine IVF practice.

### 4.10 Strengths of the study

Several strengths enhance the validity of the present investigation.

First, the study utilized a real-world retrospective cohort encompassing five years of clinical practice, providing valuable insights into the performance of AI-assisted embryo selection under routine clinical conditions.

Second, the inclusion of 300 IVF treatment cycles provided an adequate sample for evaluating associations between AI scores and reproductive outcomes.

Third, the study examined multiple clinically relevant outcomes, including implantation, clinical pregnancy, ongoing pregnancy, and live birth.

Fourth, the study incorporated multivariable statistical analyses and predictive modeling approaches consistent with contemporary recommendations for clinical prediction model development and reporting (28, 29).

Finally, the inclusion of model validation procedures enhanced the methodological rigor of the study and improved confidence in the reported predictive performance (30).

### 4.11 Limitations of the study

Compared with previously published artificial intelligence-based embryo-selection studies, the present investigation has several important shortcomings and challenges. First, this was a retrospective, single-centre study involving a relatively modest number of IVF cycles, whereas the performance of machine-learning models is strongly influenced by dataset size, patient diversity, laboratory protocols, imaging systems, and class distribution. Consequently, the observed predictive performance may partly reflect centre-specific clinical and embryological practices and may not be directly generalizable to other IVF laboratories. Second, the present model integrated demographic, clinical, embryological, and AI-derived variables to predict clinical pregnancy, while several previous studies used deep-learning architectures to directly classify embryo viability from static images or complete time-lapse sequences (8–11, 32, 33). Therefore, direct numerical comparison of accuracy or AUC values is difficult because the studies differed in their input data, outcome definitions, developmental stages, imaging platforms, train–test partitioning, and validation procedures.

Third, although the present model demonstrated favorable discrimination and calibration, internal train–test validation cannot replace independent external validation. Models developed within a single institution may be vulnerable to overfitting, data leakage, and reduced performance when applied to populations with different demographic characteristics, incubators, image-acquisition settings, culture conditions, or embryo-grading practices. Fourth, advanced deep-learning models may capture complex spatial and temporal embryo-development patterns more effectively than conventional machine-learning methods; however, they generally require larger annotated datasets, greater computational resources, and more extensive technical expertise. They may also be less interpretable, which can limit clinician confidence, regulatory evaluation, and accountability when AI recommendations conflict with embryologist assessment.

Finally, the comparison with existing literature is challenged by the absence of standardized outcome definitions and reporting frameworks. Some studies predict embryo morphology or viability, whereas others predict implantation, fetal-heart pregnancy, clinical pregnancy, or live birth. Accuracy alone is therefore insufficient for determining clinical superiority. Sensitivity, specificity, calibration, false-positive and false-negative rates, clinical usefulness, and external reproducibility should also be considered. Future studies should address these limitations through prospective multicentre validation, standardized outcome reporting, transparent description of model development, and direct comparison with experienced embryologists and currently available embryo-assessment systems.

### 4.12 Future research directions

Future investigations should focus on prospective multicenter validation of AI-based embryo selection systems across diverse patient populations and laboratory environments (22, 26). Additional research is required to determine whether AI-assisted embryo assessment translates into meaningful improvements in cumulative live birth rates and cost-effectiveness. Integration of AI-derived embryo characteristics with genomic, metabolomic, and clinical data may further enhance predictive accuracy and support personalized reproductive medicine approaches (22, 26). Emerging explainable artificial intelligence techniques may improve transparency, clinician trust, and regulatory acceptance of AI systems in reproductive healthcare (17). Furthermore, successful implementation of AI-assisted embryo selection will require consideration of ethical, operational, and healthcare delivery challenges associated with integrating machine learning technologies into routine clinical practice (27).

Practical Integration into the Routine Embryology Workflow:

To be clinically useful, AI-assisted embryo selection should be integrated into the current embryology workflow and not replace the embryologist, functioning as a decision-support tool. In a time-lapse incubator, images are taken automatically of the embryos within the incubator without disturbing the controlled culture, and are already being captured in a lab. These sequential images can be fed to the AI system, which can then generate a viability score or ranked list for each embryo even before the embryo-assessment or transfer meeting. Ranked output can then be presented with traditional data such as developmental stage, cleavage pattern, fragmentation, blastocyst expansion, inner-cell-mass grade, trophectoderm grade, fertilization method, and patient characteristics. The workflow in daily use may include a two-way automatic linkage of images of each embryo to its respective laboratory identification number, image quality checking, AI-assisted analysis and presentation of embryo scores on the embryology work station or the laboratory information system. The embryologist would take a look at the AI ranking along with the entire sequence of development and the conventional morphological evaluation. Final decisions on embryo transfer, cryopreservation, extended culture or exclusion would be made by the embryologist and clinical team in charge of the patient. The human-in-the-loop is important because the algorithm does not necessarily cover all of the unusual developmental events, poor image quality, abnormal fertilization, patient-specific factors, or laboratory conditions.

Borna et al. designed the DeepEmbryo algorithm specifically to match with the standard IVF lab process (33). They used their model to analyse three embryo images collected at 19 ± 1 hours after insemination (h.a.i.), 43 ± 1 h.a.i., and 67 ± 1 h.a.i., which is the time when sequential morphological assessment of embryos in various cleavage stages is usually performed. 252 time-lapse embryo videos were used in the study, and transfer learning was used with multiple pre-trained models of convolutional neural networks. Compared to the single image method, which predicted pregnancy outcomes with at least 60% accuracy, the multi-image approach improved the accuracy of prediction of up to 75%. The model also performed better than the five experienced embryologists that were compared in the study.

A significant practical advantage of this approach is that it does not need to be reviewed manually each time-lapse frame. Rather, meaningful frames can be automatically extracted or extracted at certain developmental moments and be given to the algorithm. Borna et al. pointed out that such imaging is already done during routine sequential embryo observations; thus, their proposed system aimed to prevent significant changes from being made to the laboratory equipment, operating procedures or embryologist workload. This approach can also help to ease the way for AI’s use in labs that carry out digital imaging occasionally rather than regularly and have no access to fully automated time-lapse analysis platforms.

The AI score obtained in the present study may be used to the same effect. Instead of reading the score as a stand-alone command, the system could classify embryos into high to low groups of predicted viability (excellent, good, moderate and poor), and identify embryos that should be subjected to further development. When the output of AI and human assessments of a case differ significantly, they should be marked as both AI and human ranking and not be automatically resolved. Additionally, the Embryologist should have the ability to overrule this suggested recommendation as well, and for the purpose of quality assurance and as a basis for model evaluation, the reason for the override should be recorded. For routine use, laboratories would need standard operating procedures for image acquisition, identification of the embryos, missing images, or poor quality images, control of model versions, user access, data security, documentation of results and algorithm failure management. Periodic monitoring of performance should be based on implantation outcomes, clinical-pregnancy outcomes, and live-birth outcomes. Local validation is extremely crucial since variations in incubator, microscope models, cell culture conditions, patient cohorts, image resolution, and laboratory processes can affect the model performance. Therefore, there should be a supervised/silent validation step when using AI-assisted ranking before it is used prospectively to assist decisions in embryo-selection.

## 5 Conclusion

The present study demonstrated that AI-based non-invasive embryo selection was strongly associated with improved IVF outcomes and provided significant predictive value beyond conventional embryo-grading methods. Higher AI embryo scores were independently associated with increased clinical pregnancy rates, while the developed predictive model exhibited excellent discrimination and calibration. These findings support the potential role of AI-assisted embryo assessment in improving the objectivity, accuracy, and individualization of embryo-selection decisions. However, because the model was developed retrospectively using data from a single clinical center, its performance cannot yet be assumed to generalize across different patient populations, laboratory protocols, imaging systems, incubators, or clinical settings. Future studies must therefore include prospective evaluation and rigorous external validation in independent, geographically and clinically diverse fertility centers. Such multicenter validation should assess discrimination, calibration, clinical utility, reproducibility, and effects on implantation, cumulative live-birth rates, workflow, and cost-effectiveness. Successful real-world validation is essential before the model can be recommended for routine clinical decision-making in IVF laboratories.

## Data Availability

The original contributions presented in the study are included in the article/supplementary material. Further inquiries can be directed to the corresponding author.
